# HIV-positive Nigerian adults harbour significantly higher serum lumefantrine levels than HIV negative individuals seven days after treatment for *Plasmodium falciparum* infection

**DOI:** 10.1186/1475-2875-11-S1-P16

**Published:** 2012-10-15

**Authors:** Ifevinwa Chijioke-Nwauche, Albert van Wyk, Chijioke Nwauche, Harparkash Kaur, Colin J Sutherland

**Affiliations:** 1 Department of Clinical Pharmacy & Management, Faculty of Pharmaceutical Sciences, University of Port Harcourt, Nigeria; 2 Department of Immunology & Infection, Faculty of Infectious & Tropical Diseases, LSHTM, London, UK; 3 Department of Haematology Blood Transfusion and Immunology, College of Health Sciences, University of Port Harcourt, Nigeria; 4 Centre for Malaria Research & Phytomedicine, University of Port Harcourt, Nigeria; 5 Department of Disease Control, Faculty of Infectious & Tropical Diseases, LSHTM, London, UK

## Background

Management of co-infection with malaria and HIV is a major challenge to public health and yet potential drug-drug interactions between antimalarial and antiviral regimens have not been adequately investigated in people with both infections. Both of the constituent components of artemether-lumefantrine (AL) the first-line regimen for malaria treatment in Nigeria, and nevirapine, a major component of highly active antiretroviral therapy, are drugs metabolised by the cytochrome P450 3A4 isoenzyme system, known to be induced by nevirapine. We examined potential interactions between lumefantrine and nevirapine in 68 HIV-positive and 99 HIV-negative adults, all of whom were diagnosed with asymptomatic *Plasmodium falciparum* infections by microscopy. *Post hoc* PCR analysis confirmed the presence of *P. falciparum* in only a minority of participants.

## Materials and methods

68 out of 80 attendees at the HIV clinics tested were identified as positive for *P. falciparum* and returned for day 7 follow-up (85%). None of these individuals reported concurrent symptoms suggestive of clinical malaria. 126 additional volunteers agreed to have a rapid HIV test performed, of which 99 were found to be both negative for HIV and infected with *P. falciparum.* None of these individuals were symptomatic. All 167 participants were treated with a full adult course of AL, and followed up on days 3, 7 and 28 for repeat blood sampling. We recorded and examined the distribution of lumefantrine concentration at day 7 in all study participants.

## Results

Using the PCR data as a more reliable test for parasite carriage, we found weak evidence that HIV positive people were more likely to be parasitaemic at day 0 (OR 2.05, 95% C.I. 0.917 - 4.60; P = 0.054), which may reflect slightly higher parasite densities in this group. HIV-positive subjects were not significantly more likely to be PCR positive for *P. falciparum* at day 3 and/or day 28 after AL treatment than were HIV negative individuals (OR 1.75, 95% C.I. 0.776 - 3.95; P = 0.141).

HIV status, and thus nevirapine use, was found to have a significant effect on the concentration of lumefantrine 7 days after treatment (Wilcoxon ranksum test z = -3.270, P=0.0011), with a median concentration in the HIV negative group of 2.75µM (IQR 1.03 - 4.31), and in the HIV positive group of 3.55µM (IQR 2.07 - 5.37. There was a significant association between HIV status and lumefantrine concentration at 7 days post AL treatment (z = -2.830, P=0.0046). Day 7 capillary blood levels of lumefantrine were significantly higher in nevirapine-treated HIV positive participants than in 99 HIV negative controls (P=0.0011). Higher day 7 levels of lumefantrine were not associated with lower risk of persistent PCR-detectable parasitaemia at day 3 post-treatment.

## Conclusion

Nevirapine increases peripheral lumefantinre levels in AL-treated adult African malaria patients. Preliminary data suggest that higher lumefantrine concentrations do not provide any parasitological benefit to nevirapine-treated HIV patients.

